# PIPKIγ Regulates Focal Adhesion Dynamics and Colon Cancer Cell Invasion

**DOI:** 10.1371/journal.pone.0024775

**Published:** 2011-09-12

**Authors:** Zhaofei Wu, Xiang Li, Manjula Sunkara, Heather Spearman, Andrew J. Morris, Cai Huang

**Affiliations:** 1 Markey Cancer Center, University of Kentucky, Lexington, Kentucky, United States of America; 2 Division of Cardiovascular Medicine, University of Kentucky, Lexington, Kentucky, United States of America; 3 Department of Internal Medicine, University of Kentucky, Lexington, Kentucky, United States of America; 4 Department of Molecular and Biomedical Pharmacology, University of Kentucky, Lexington, Kentucky, United States of America; Kings College London, United Kingdom

## Abstract

Focal adhesion assembly and disassembly are essential for cell migration and cancer invasion, but the detailed molecular mechanisms regulating these processes remain to be elucidated. Phosphatidylinositol phosphate kinase type Iγ (PIPKIγ) binds talin and is required for focal adhesion formation in EGF-stimulated cells, but its role in regulating focal adhesion dynamics and cancer invasion is poorly understood. We show here that overexpression of PIPKIγ promoted focal adhesion formation, whereas cells expressing either PIPKIγ^K188,200R^ or PIPKIγ^D316K^, two kinase-dead mutants, had much fewer focal adhesions than those expressing WT PIPKIγ in CHO-K1 cells and HCT116 colon cancer cells. Furthermore, overexpression of PIPKIγ, but not PIPKIγ^K188,200R^, resulted in an increase in both focal adhesion assembly and disassembly rates. Depletion of PIPKIγ by using shRNA strongly inhibited formation of focal adhesions in HCT116 cells. Overexpression of PIPKIγ^K188,200R^ or depletion of PIPKIγ reduced the strength of HCT116 cell adhesion to fibronection and inhibited the invasive capacities of HCT116 cells. PIPKIγ depletion reduced PIP_2_ levels to ∼40% of control and PIP_3_ to undetectable levels, and inhibited vinculin localizing to focal adhesions. Taken together, PIPKIγ positively regulates focal adhesion dynamics and cancer invasion, most probably through PIP_2_-mediated vinculin activation.

## Introduction

Focal adhesions (FAs, also called cell-matrix adhesions) are specific types of large macromolecular assemblies at the ventral surface of cells, functioning as both mechanical machineries and regulatory signaling hubs [1], [2]. Temporal and spatial regulation of focal adhesion assembly/disassembly is required for cell migration [3]. During cell migration, nascent focal adhesions (also called focal complexes) are formed to stabilize lamellipodia at the front of cells while focal adhesions are dissolved at the trailing edges of cells [4], [5].

Focal adhesion assembly and disassembly are also implicated in cancer invasion, a prerequisite for metastasis. DRR (Down-Regulated in Renal cell carcinoma) associates with actin and microtubules and stimulates glioma invasion by promoting focal adhesion disassembly [6]. The actin cross-linking protein filamin A suppresses focal adhesion disassembly and breast cancer cell invasion [7]. Rho/Rock signaling promotes tumor cell migration and invasion by regulating focal adhesion dynamics through caveolin-1 phosphorylation [8]. FAK also promotes focal adhesion disassembly and cancer invasion [9], [10], [11]. Focal adhesion dynamics and signaling pathways that regulate this process could be therefore an attractive target for cancer therapy.

Many molecules have been shown to regulate focal adhesion dynamics. FAK regulates focal adhesion turnover [9], [12], probably through a dynamin and microtubule-dependent pathway [13]. Paxillin, a focal adhesion adaptor, also modulates focal adhesion dynamics by JNK and PAK-mediated phosphorylation [14], [15]. Talin activates integrins and initiates focal adhesion formation [16], [17], [18], whereas cleavage of talin by calpain mediates focal adhesion disassembly [19]. Calpain also cleaves FAK and paxillin to modulate focal adhesion dynamics [20], [21]. We have shown that Smurf1-mediated ubiquitination of the talin head domain, one of the two cleavage products, plays an important role in focal adhesion disassembly and cell migration [22]. ACF7, a microtubule and filamentous actin binding protein, regulates focal adhesion assembly/disassembly through its ATPase activity [23]. However, the molecular mechanisms that control focal adhesion assembly/disassembly are not fully understood.

Phosphatidylinositol phosphate kinase type I γ (PIPKIγ) is an enzyme that catalyzes ATP-dependent phosphorylation of phosphatidylinositol 4-phosphate (PI(4)P) to generate PI(4,5)P_2_, which regulates a variety of biological processes, including focal adhesion formation [24]. PIPKIγ has a well-conserved kinase catalytic domain at the central region [25]. Within the catalytic domain, there is a subdomain called the activation loop, which determines site of phosphorylation on the inositol ring of the substrate. PIPKIγ strongly interacts with talin and competes for talin binding with the β integrin tail [26], [27]. It is localized at adherens junctions in epithelial cells [28], [29]. It has been reported that PIPKIγ is required for focal adhesion formation in migratory cells [30]. However, the precise roles of the lipid kinase activities of PIPKIγ in focal adhesion dynamics are not defined.

In the present study, we investigated the requirement for the lipid kinase activity of PIPKIγ in focal adhesion dynamics and colon cancer invasion. Our results identify an essential role of PIPKIγ in focal adhesion assembly/disassembly and cancer invasion.

## Results

### PIPKIγ promotes focal adhesion formation

**Figure 1:**
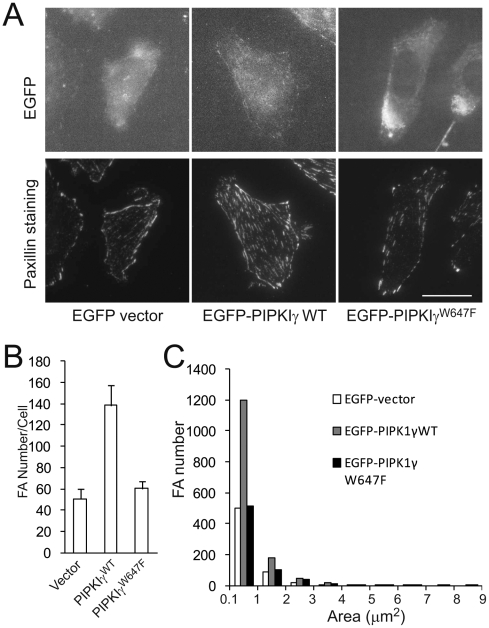
PIPKIγ promoted focal adhesion formation in CHO-K1 cells. (**A**) TIRF images of CHO-K1 cells expressing EGFP, EGFP-PIPKIγ WT or EGFP-PIPKIγ^W647F^. Cells were transiently transfected with pEGFP-vector, -PIPKIγ WT or -PIPKIγ^W647F^, and then stained for paxillin. Scale bar, 20 µm. (**B**) Expression of PIPKIγ but not PIPKIγ^W647F^ caused an increase in focal adhesion number (n = 15, error bar = mean ± s.e.m; Vector *vs* WT, P<0.001; WT *vs* W647F, P<0.001; Vector *vs* W647F, P>0.05). (**C**) Area distribution of focal adhesions in cells expressing EGFP, EGFP-PIPKIγ WT or EGFP-PIPKIγ^W647F^.

To examine a possible role for PIPKIγ in focal adhesion formation, CHO-K1 cells were transfected with EGFP-PIPKIγ or EGFP vector, respectively. The cells were re-plated on fibronectin, fixed with paraformaldehyde, incubated with an anti-paxillin monoclonal antibody, and then stained with Dylight 549 conjugated goat anti-mouse IgG. Focal adhesions were lined up around the edges of the EGFP vector-transfected cells, with few focal adhesions in the centers of the cells, whereas PIPKIγ expression dramatically stimulated focal adhesion formation in the centers of the cells (Fig. 1A). Over-expression of PIPKIγ stimulates an increase in focal adhesion numbers (Fig. 1B) and the increase was mainly contributed by small focal adhesions (<3 µm^2^) (Fig. 1C). The stimulation of focal adhesion formation by PIPKIγ relies on its interaction with talin, because PIPKIγ^W647F^, a mutant that is deficient in the interaction with talin, was not able to promote focal adhesion formation (Fig. 1A, B, & C).

### PIPKIγ kinase activity is required for stimulation of focal adhesion formation

**Figure 2:**
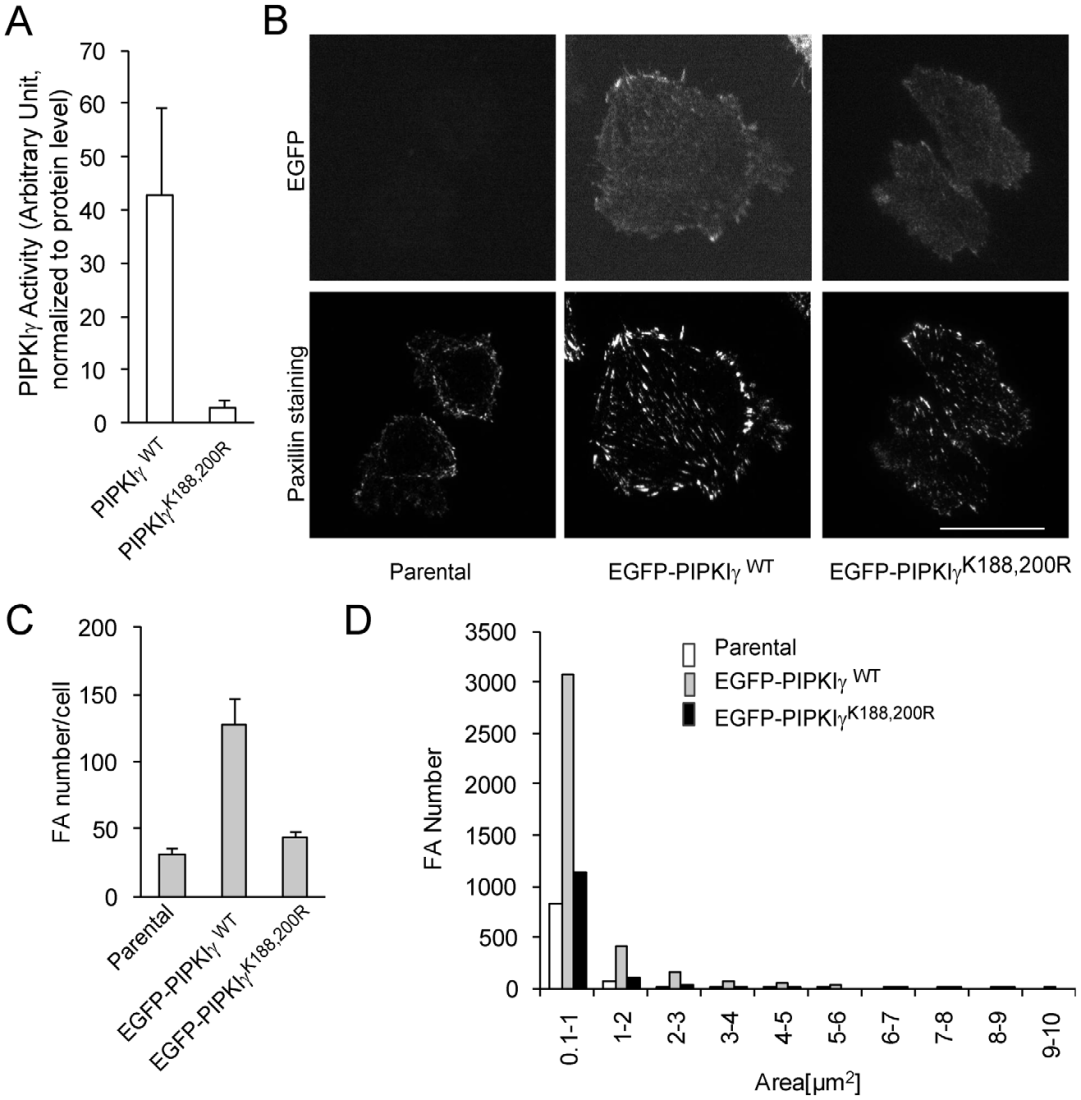
Mutation at the kinase domain of PIPKIγ abolished its activity and ability to promote focal adhesion formation in CHO-K1 cells. (A) Analysis of the activities of PIPKIγ and PIPKIγ^K188,200R^. (B) TIRF images of CHO-K1 cells that express PIPKIγ or PIPKIγ^K188,200R^ and the parental CHO-K1 cells. The parental cells and cells expressing EGFP-PIPKIγ WT or -PIPKIγ^K188, 200R^ were stained for paxillin. Scale bar, 20 µm. (C) Quantitative analysis of focal adhesion numbers (per cell) in the parental cells and cells that express PIPKIγ or PIPKIγ^K188,200R^ (n = 30, error bar = mean ± s.e.m; parental vs PIPKIγ, P<0.001; parental vs PIPKIγ^K188,200R^, P>0.05). (D) Area distribution of focal adhesions in the parental cells and cells expressing PIPKIγ and PIPKIγ^K188,200R^.

PI(4,5)P_2_ binds talin and strengthens the interaction between talin and the β integrin tail, stimulating integrin clustering [31]. PI(4,5)P_2_ also binds vinculin and unmasks the actin and talin binding sites on vinculin, promoting focal adhesion formation [32]. Therefore, PIPKIγ-stimulated PI(4,5)P_2_ synthesis could be essential for promoting focal adhesion formation. To test this hypothesis, we mutated two lysine residues, K188 and K200, to arginine residues within the ATP-binding site of PIPKIγ. The mutant PIPKIγ^K188,200R^ and the WT were purified from CHO-K1 cells and the kinase activities were examined *in vitro* by using mass spectrometry to quantitate production of PI(4,5)P_2_ from PI(4)P. As shown in Fig. 2A, mutation at K188 and K200 reduced kinase activity by 95%. EGFP-tagged mutant PIPKIγ^K188,200R^ and WT PIPKIγ were stably expressed in CHO-K1 cells, and their effects on focal adhesion formation were examined after paxillin staining. Cells that stably express WT PIPKIγ formed focal adhesions around the edges and in the centers of cells, whereas cells that express PIPKIγ^K188,200R^, similar to parental cells, possessed small focal adhesions, most of which were around the edges of the cells, and had a defect in spreading (Fig. 2B). Quantitative analysis indicated that PIPKIγ increased focal adhesions by more than 2 fold, whereas PIPKIγ^K188,200R^ did not significantly promote focal adhesion formation (Fig. 2C&D). These results indicate that PIPKIγ activity is essential for focal adhesion formation in CHO-K1 cells.

To further verify the requirement of PIPKIγ activity for focal adhesion formation, we tested the capacity of another kinase dead mutant, PIPKIγ^D316K^ [33], to promote focal adhesion formation as described above. As shown in Fig. S1, the average focal adhesion number (per cell) in cells expressing PIPKIγ^D316K^ was approximately 45, which is approximately the same as that in cells expressing EGFP vector (Fig. 1). The focal adhesions in cells expressing PIPKIγ^D316K^ accumulated at the edges of the cells, with very few focal adhesions in the center of the cells. This result confirms the essential role of PIPKIγ activity in focal adhesion formation.

### The activity of PIPKIγ is essential for its promoting focal adhesion dynamics

**Figure 3:**
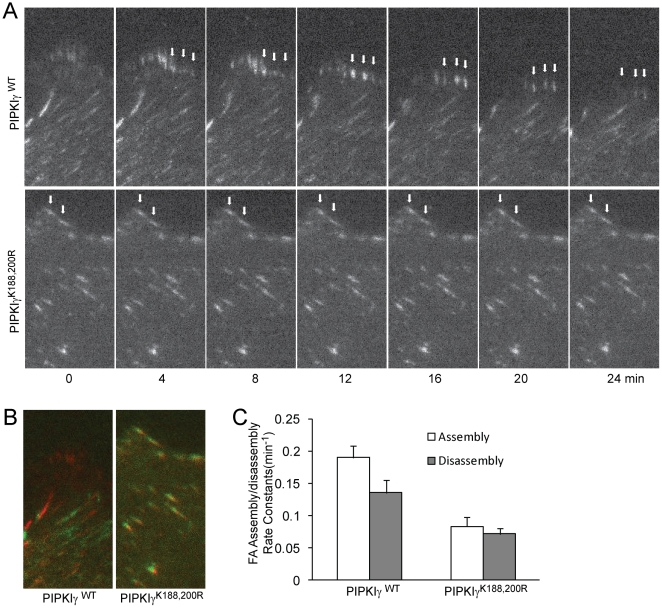
PIPKIγ WT but not PIPKIγ^K188,200R^ promoted focal adhesion assembly and disassembly in CHO-K1 cells. (A) CHO-K1 cells stably expressing PIPKIγ or PIPKIγ^K188,200R^ were transiently transfected with mDsRed-paxillin. The cells were plated on fibronectin and the dynamics of paxillin was analysed using time-lapse TIRF microscopy. Arrowheads point to dynamic (upper panels) and stable (lower panels) focal adhesions. (B) The overlay of 0 and 24 min in “A”. (C) Quantification of the focal adhesion assembly and disassembly rate constants in cells that express PIPKIγ or PIPKIγ^K188,200R^. Quantifications are expressed as mean ± s.e.m. of 50 focal adhesions from 10 cells. Assembly, WT vs K188,200R, P<0.00005; disassembly, p<0.005.

To examine whether PIPKIγ lipid kinase activity is required for focal adhesion dynamics, CHO-K1 cells that stably express EGFP-PIPKIγ or -PIPKIγ^K188,200R^ were transfected with mDsRed-paxillin and then plated on MatTek dishes (with a glass coverslip at the bottom) precoated with fibronectin (5 µg/ml) and grown for 3 hr. TIRF images of mDsRed-paxillin were taken using a Nikon TIRF microscope and the temperature was maintained at 37°C using an INU-TIZ-F1 microscope incubation system (Tokai Hit). Images were recorded at 1-min intervals for a 120 min period. Focal adhesion assembly and disassembly rate constants were calculated as described previously [22]. The FA assembly and disassembly rate constants in cells expressing PIPKIγ^K188,200R^ were 0.083±0.014 and 0.072±0.010 min^−1^, respectively, which are similar to those in normal CHO-K1 cells that we reported previously [22], whereas FA assembly and disassembly rate constants were 0.190±0.019 and 0.136±0.014 min^−1^, respectively, in cells expressing WT PIPKIγ (Fig. 3). This result indicates that the inositol lipid kinase activity of PIPKIγ is required for its stimulation of focal adhesion assembly and disassembly.

### An essential role for PIPKIγ in focal adhesion formation in colon cancer cells

**Figure 4:**
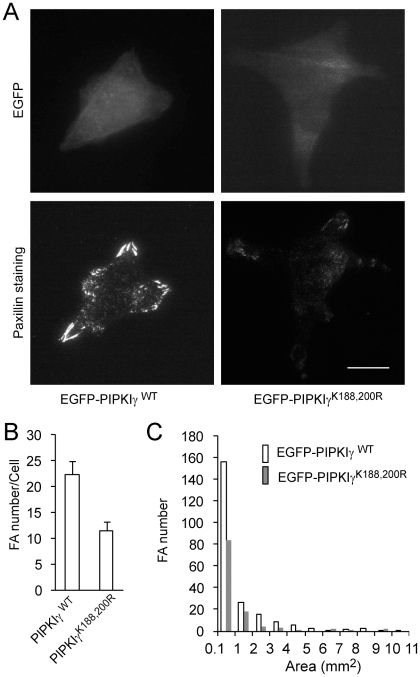
PIPKIγ^K188,200R^ was incapable of mediating focal adhesion formation in HCT116 cells. (A) TIRF images of CHO-K1 cells expressing PIPKIγ or PIPKIγ^K188,200R^. Cells that stably express EGFP-PIPKIγ WT or -PIPKIγ^K188, 200R^ were stained for paxillin. Scale bar, 20 µm. (B) PIPKIγ^K188, 200R^ failed to stimulate an increase in focal adhesion number (n = 10, error bar = mean ± s.e.m; paired t-test, P<0.005). (C) Area distribution of focal adhesions in cells expressing PIPKIγ and PIPKIγ^K188,200R^.

Focal adhesions have been implicated in regulating cancer invasion, while the role of focal adhesions in colon cancer cells has not been well defined. To determine if the activity of PIPKIγ influences focal adhesion formation in colon cancer cells, HCT116 cells that stably express EGFP-PIPKIγ or -PIPKIγ^K188,200R^ were plated on fibronectin and stained for paxillin. As shown in Fig. 4A, most of the focal adhesions were located to the peripheral region, and HCT116 cells expressing PIPKIγ^K188,200R^ had much fewer focal adhesions than those expressing the WT enzyme. The average focal adhesion number in cells expressing WT PIPKIγ was 22.5/cell, whereas that in cells expressing PIPKIγ^K188,200R^ was 11.5/cell, both of which were fewer than those observed in CHO-K1 cells (Fig. 4B). In addition, PIPKIγ^K188,200R^ had more effect on the smaller focal adhesions than the larger ones (Fig. 4C).

**Figure 5:**
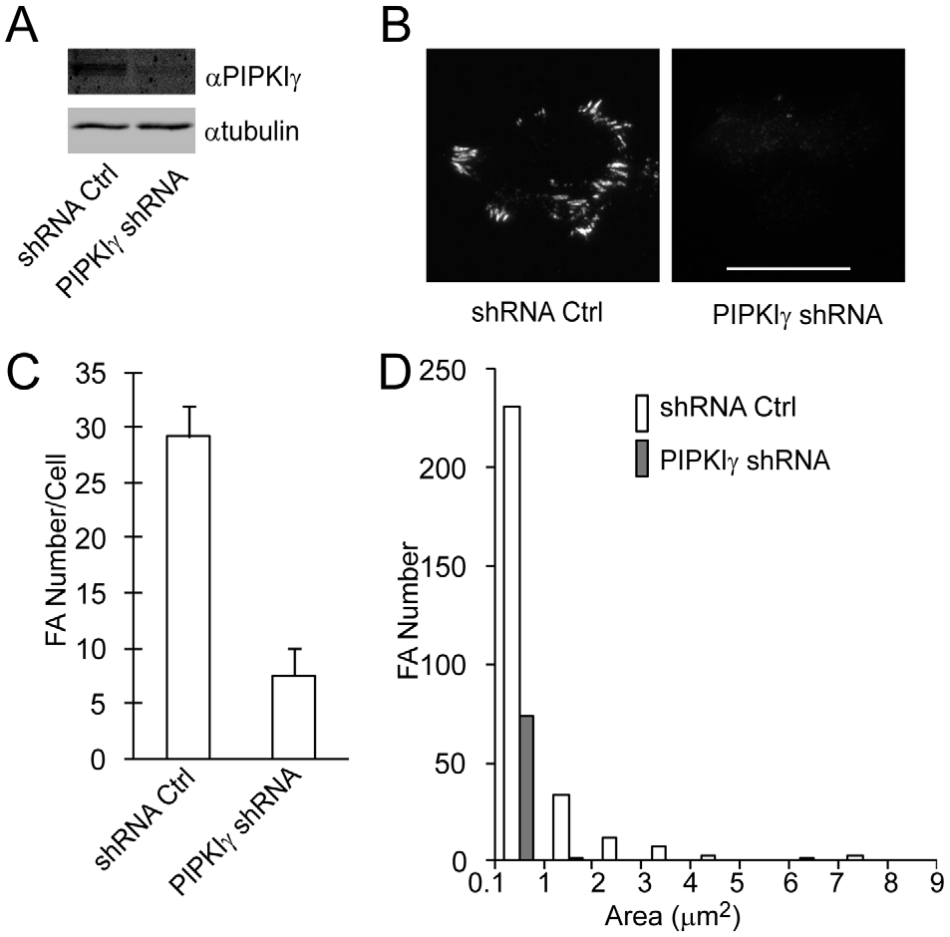
PIPKIγ is essential for focal adhesion formation in HCT116 cells. (A) Expression of PIPKIγ in cells stably expressing shRNA control and PIPKIγ shRNA. (B) TIRF images of HCT116 cells that stably express control and PIPKIγ shRNA. Cells were infected with lentiviral particles carrying control or PIPKIγ shRNA, selected with puromycin, and stained for paxillin. Scale bar, 20 µm. (C) PIPKIγ depletion inhibited focal adhesion formation in HCT116 cells. (n = 10, error bar = mean ± s.e.m; paired t-test, P<0.0001) (D) Area distribution of focal adhesions in cells expressing control and PIPKIγ shRNA.

To test whether PIPKIγ is essential for focal adhesion formation in HCT116 cells, HCT116 cells were infected with recombinant lentiviruses that express PIPKIγ shRNA or shRNA control and were selected with puromycin. As shown in Fig. 5A, expression of PIPKIγ shRNA resulted in dramatic reduction in the endogenous PIPKIγ level of HCT116 cells. The cells were then plated on fibronectin and stained for paxillin. Surprisingly, PIPKIγ knockdown almost abolished focal adhesion formation in HCT116 cells (Fig. 5B). PIPKIγ depletion reduced focal adhesion number by about 74% (Fig. 5C). Different from PIPKIγ^K188,200R^, PIPKIγ shRNA had more effect on the larger focal adhesions than the smaller ones (Fig. 5D). These results indicate an essential role of PIPKIγ in focal adhesion formation in HCT116 colon cancer cells. This dramatic effect of PIPKIγ knockdown did not occur in MDA-MB-231 human breast cancer cells, where PIPKIγ knockdown only partially inhibited focal adhesion formation (data not shown).

### PIPKIγ positively modulates adhesion strength of colon cancer cells to fibronectin

**Figure 6:**
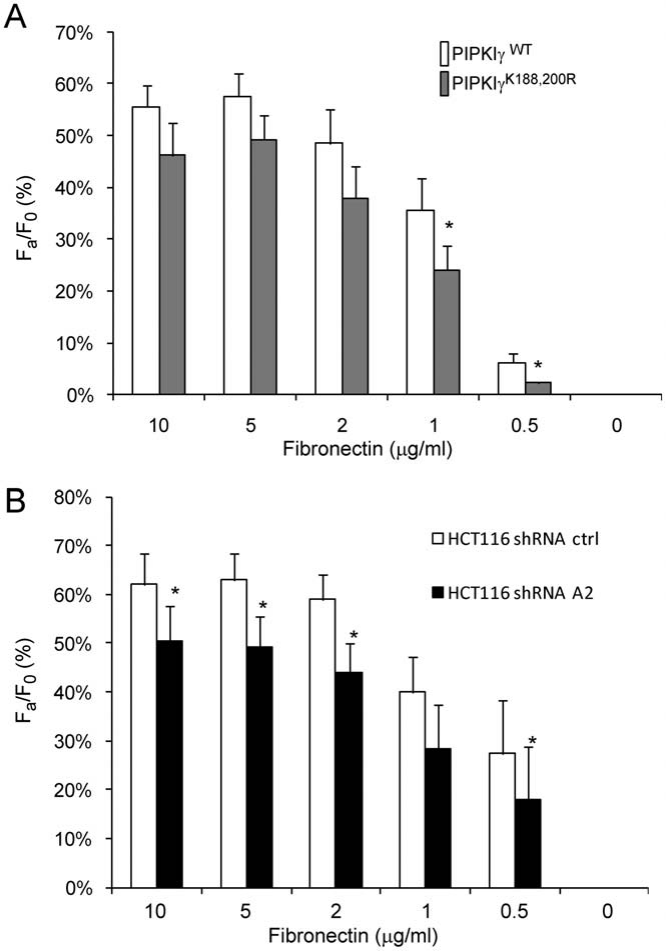
Either expression of PIPKIγ^K188,200R^ or depletion of PIPKIγ impaired the adhesion strength of HCT116 cells on fibronectin. (A) Expression of PIPKIγ^K188,200R^ inhibited HCT116 cells adhering to low concentrations of fibronectin. The cells stably expressing PIPKIγ or PIPKIγ^K188,200R^ were incubated with Calcein-AM and plated on a 96-well plate coated with fibronectin for centrifugation assays. (B) Depletion of PIPKIγ inhibited HCT116 cells adhering to fibronectin. The cells that stably express control and PIPKIγ shRNA were incubated with Calcein-AM and plated on a 96-well plate coated with fibronectin for centrifugation assays. F_a_/F_0_ =  (fluorescence after centrifugation)÷(fluorescence before centrifugation).

To examine whether the activity of PIPKIγ regulates cell adhesion strength on fibronectin, HCT116 cells that stably express EGFP-PIPKIγ or -PIPKIγ^K188,200R^ were stained with calcein-AM and seeded on a 96-well plate that was pre-coated with different concentrations of fibronectin. The calcein fluorescence was read, and then the plate was inverted and centrifuged at 150×g for 5 min. The calcein fluorescence was read again. As shown in Fig. 6A, the cell adhesion strength difference between the cells expressing PIPKIγ^K188,200R^ and the WT was not significant at high concentrations of fibronection. However, at low concentrations of fibronectin, the cells expressing PIPKIγ^K188,200R^ had significantly lower adhesion strength as compared to those expressing the WT. PIPKIγ knockdown also caused a significant decrease in cell adhesion strength in HCT116 cells (Fig. 6B). These results indicate that PIPKIγ activity regulates cell adhesion strength in colon cancer cells.

### PIPKIγ is essential for invasion but not the migration of colon cancer cells

It has been reported that PIPKIγ plays a role in cell migration. To test whether PIPKIγ regulates the migration of HCT116 colon cancer cells, we employed time-lapsed wound-healing assays to examine the migration of HCT116 cells expressing EGFP-PIPKIγ and –PIPKIγ^K188,200R^, respectively, in the presence of HGF. As shown in Fig. S2 A&B, cells expressing PIPKIγ^K188,200R^ migrated slightly faster than those expressing the WT enzyme as measured by time-lapse wound-healing assays. Also, depletion of PIPKIγ had no significant effect on the migration of HCT116 cells in transwell assays (Fig. S2 C&D). These results suggest that PIPKIγ activity is not essential for the migration of HCT116 colon cancer cells.

**Figure 7:**
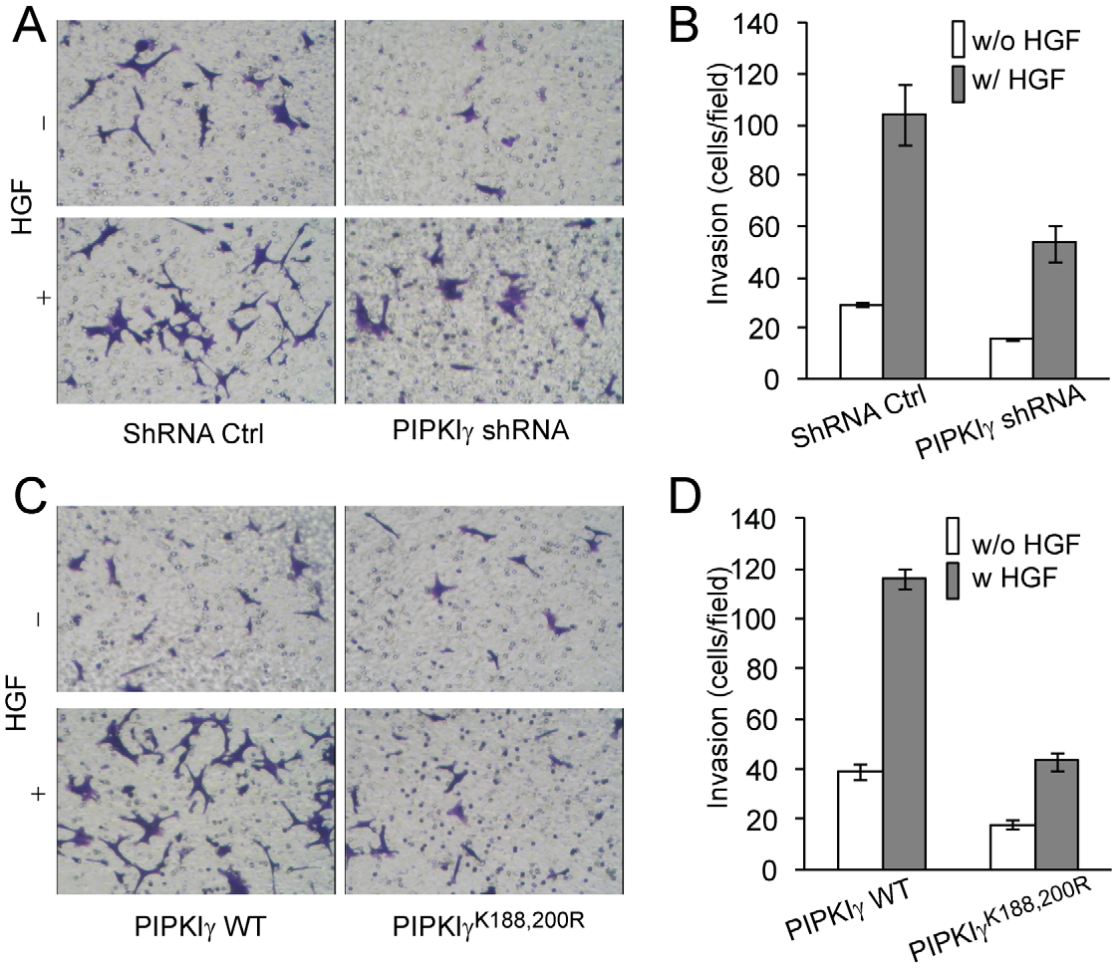
Either depletion of PIPKIγ or expression of PIPKIγ^K188,200R^ inhibited the invasion of HCT116 cells. (A) Depletion of PIPKIγ by using shRNA inhibited the invasion of HCT116 cells. The invasive capacities of cells stably expressing shRNA control or PIPKIγ shRNA were examined in the absence and presence of HGF (50 ng/ml) by Matrigel-based invasion assays. (B) Quantification of the invasion of HCT116 cells that stably express shRNA control or PIPKIγ shRNA. n = 5, error bar = mean ± s.e.m; ctrl vs PIPKIγ, p<0.01; ctrl HGF vs PIPKIγ HGF, p<0.01. (C) Expression of PIPKIγ^K188,200R^ inhibited the invasion of HCT116 cells. The cells that stably express EGFP-PIPKIγ or –PIPKIγ^K188,200R^ were tested for invasive capacities in the absence and presence of HGF. (D) Quantification of the invasion of HCT116 cells that stably express EGFP-PIPKIγ or –PIPKIγ^K188,200R^. n = 3, error bar = mean ± s.e.m; WT vs K188,200R, P<0.01; WT HGF vs K188,200R HGF, P<0.005.

To test the role of PIPKIγ in cancer invasion, the invasion of HCT116 cells that stably express PIPKIγ shRNA or a shRNA control in the absence and presence of HGF was examined by using Matrigel invasion assays. As shown in Fig. 7 (A&B), PIPKIγ knockdown resulted in significant reduction in the invasion of HCT116 cells either in the absence or in the presence of HGF. The basal and HGF-stimulated invasive capacities of HCT116 cells stably expressing PIPKIγ^K188,200R^ are also significantly lower than those of the cells expressing the WT enzyme (Fig. 7C&D). These results indicate that PIPKIγ positively regulates the invasion of HCT116 colon cancer cells.

### PIPKIγ regulates focal adhesion formation through activating vinculin by PI(4,5)P_2_

**Figure 8:**
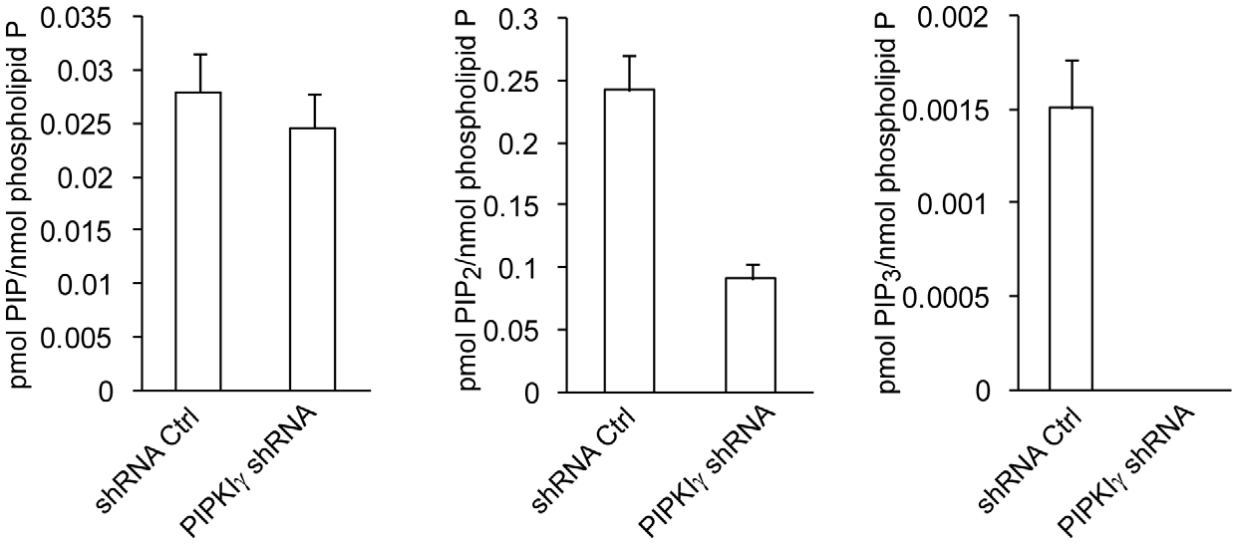
PIPKIγ is a major enzyme responsible for PIP2 and PIP3 production in HCT116 cells. Polyphosphoinositides from cells stably expressing shRNA control or PIPKIγ shRNA were extracted and derivatized using trimethylsilyl diazomethane, and then were measured using mass spectrometry. N = 4, error bar = mean ± s.e.m; PIP, P>0.05; PIP2, P = 0.0034; PIP3, P = 0.001.

To dissect the mechanism by which PIPKIγ regulates focal adhesion formation, we set out to analyze the levels of polyphosphoinositides in HCT116 cells that express PIPKIγ shRNA or a shRNA control by using mass spectrometry. Depletion of PIPKIγ in HCT116 cells had no significant effect on the level of PIP, but caused significant reduction in PIP_2_ level (note that these methods cannot distinguish positional enantiomers of these lipids so it is possible that PI(3,4)P_2_ may contribute significantly to residual levels of PIP_2_ in these cells). Interestingly, in support of this idea knockdown of PIPKIγ reduced PIP_3_ levels below the detection limit of our assay (Fig. 8). These results indicate that PIPKIγ is a key enzyme responsible for the production of PI(4,5)P_2_ and PI(3,4,5)P_3_ in HCT116 cells.

To see whether PI(3,4,5)P_3_ is essential for focal adhesion formation in colon cancer cells, HCT116 cells were treated with LY294002, a specific inhibitor of phosphatidylinositol 3-kinase, and focal adhesion formation in these cells was examined after paxillin staining. LY294002 (20 µM) had no significant effect on focal adhesion formation (data not shown). Taken together with the above observations, this finding indicates that PI(4,5)P_2_, but not PI(3,4,5)P_3_, is required for focal adhesion formation.

**Figure 9:**
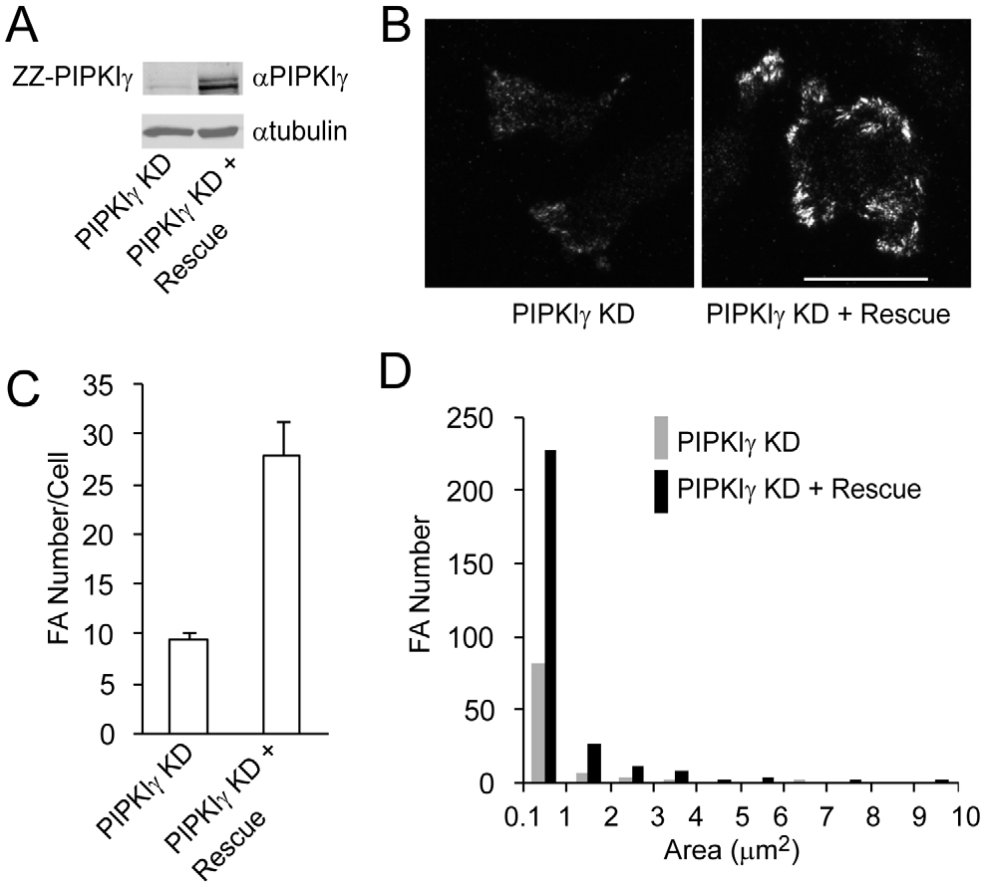
PIPKIγ is required for vinculin localizing to focal adhesions in HCT116 cells. (A) Re-expression of rescue ZZ-tagged PIPKIγ in cells that PIPKIγ has been knocked down (KD) by stably expressing PIPKIγ shRNA. The PIPKIγ-KD cells were infected with retroviral particles carrying rescue ZZ-tagged PIPKIγ selected with neomycin. ZZ-PIPKI was detected by Western blotting with an anti-PIPKIγ polyclonal antibody. (B) TIRF images of PIPKIγ-KD cells and the KD cells that express rescue PIPKIγ Cells were plated on glass-bottomed dishes pre-coated with fibronectin (5 µg/ml), fixed and stained for vinculin. Scale bar, 20 µm. (C) PIPKIγ rescue restored focal adhesion formation in PIPKIγ-KD cells. (n = 11, error bar = mean ± s.e.m; paired t-test, P<0.0001) (D) Area distribution of focal adhesions in PIPKIγ-KD cells and the KD cells expressing rescue PIPKIγ.

PI(4,5)P_2_ binds and activates vinculin and is implicated in regulating focal adhesion formation [32]. If PIPKIγ-mediated production of PI(4,5)P_2_ is essential for focal adhesion formation in HCT116 cells, depletion of PIPKIγ would reduce PI(4,5)P_2_ levels and prevent vinculin from localizing to focal adhesions. To test this hypothesis, endogenous PIPKIγ-depleted cells were infected with retroviruses expressing a codon-modified PIPKIγ (rescue) (Fig. 9A), and the cells were stained for vinculin. Vinculin was rarely localized to focal adhesions in PIPKIγ-depleted HCT116 cells, whereas re-expression of PIPKIγ in PIPKIγ-depleted cells resulted in a dramatic increase in focal adhesion-localized vinculin (Fig. 9B). Quantitative analysis indicated that PIPKIγ rescue restored focal adhesion formation in PIPKIγ-depleted cells (Fig. 9C&D), as compared to the data in Fig. 5. These data suggest that PIPKIγ may regulate focal adhesion formation through PI(4,5)P_2_-mediated vinculin activation.

## Discussion

Besides serving as the precursor of other second messengers, PI(4,5)P_2_ itself binds many cytoskeletal and focal adhesion proteins and is believed to be a key regulator of focal adhesion dynamics [34]. PI(4,5)P_2_ binds vinculin to unmask the talin-binding sites on vinculin [32]; it also binds talin thus stabilizing talin-integrin interactions [35]. PIPKIγ is thought to be the enzyme that generates PI(4,5)P_2_ spatially and temporally for focal adhesion formation during cell migration [27], [34]. On the other hand, PI(4,5)P_2_ has not been detected at focal adhesions and the role of PIPKIγ in regulating focal adhesion dynamics is controversial [36]. PIPKIγ has been shown to be required for focal adhesion formation during EGF-stimulated cell migration [30], whereas it has also been reported that expression of PIPKIγ caused cell rounding and focal adhesion disassembly [26].

We show here that expression of PIPKIγ at low levels in CHO-K1 cells stimulated focal adhesion formation (Fig. 1), whereas kinase-dead mutants, PIPKIγ^K188,200R^ and PIPKIγ^D316K^ failed to promote focal adhesion formation (Fig. 2, Fig. 4 and Fig. S1). Furthermore, PIPKIγ knockdown almost completely abolished focal adhesion formation in HCT116 colon cancer cells (Fig. 5). In addition, expression of PIPKIγ promoted focal adhesion assembly and also disassembly rates, while PIPKIγ^K188,200R^ was unable to do so (Fig. 3). These results identify an essential role of PIPKIγ in regulating focal adhesion dynamics.

Although direct evidence is lacking, PI(4,5)P_2_ has been well implicated in regulating integrin activation. PI(4,5)P_2_ binds talin and blocks its self-inhibition (head-tail interaction) thus promoting its interaction with the β integrin tail [35]. It also stimulates integrin clustering [31]. The increase in both talin-integrin interaction and integrin clustering should stimulate integrin activation. We found that HCT116 cells expressing PIPKIγ^K188,200R^ have significantly lower adhesion strength as compared to those expressing the WT, and depletion of PIPKIγ by shRNA also caused a significant decrease in cell adhesion strength in HCT116 cells (Fig. 6), suggesting that PIPKIγ may modulate integrin activation in colon cancer cells.

It has been reported that PIPKIγ is required for EGF-stimulated migration of MDA-MB-231 human breast cancer cells and HeLa human ovarian cancer cells [30], [37]. However, our results here show that neither expressing PIPKIγ^K188,200R^, a kinase-dead mutant, nor depletion of PIPKIγ inhibit the migration of HCT116 cells (Fig. S2). The MDA-MB-231 and HeLa cells migrate much faster than HCT116 cells, suggesting that PIPKIγ is essential for fast-moving cells, but not for slow-migrating cells like HCT116 cells. This is supported by our unpublished result that PIPKIγ^K188,200R^ dramatically inhibits the migration of Clone A cells, a fast-moving colon cancer cell line.

On the other hand, PIPKIγ seems to be required for the invasion of both fast- (such as MDA-MB-231) and slow-invading (such as HCT116) cancer cells. Depletion of PIPKIγ has been shown to inhibit EGF-stimulated invasion of MDA-MB-231 cells [37], and either expressing PIPKIγ^K188,200R^ or the depletion of PIPKIγ inhibits the invasion of HCT116 cells (Fig. 7). These results indicate an essential role of PIPKIγ in controlling cancer invasion.

Previous studies have focused on the role of focal adhesion disassembly in regulating cancer invasion. DRR promotes glioma invasion by promoting focal adhesion disassembly [6]. Filamin A suppresses breast cancer cell invasion by inhibiting focal adhesion disassembly [7]. FAK also promotes focal adhesion disassembly and cancer invasion [9]. However, our results show that both expression of PIPKIγ^K188,200R^, the kinase-dead mutant, and depletion of PIPKIγ impair focal adhesion formation, accompanying the inhibition of the invasion of HCT116 (Fig. 4, 5 and 7). In addition, PIPKIγ stimulates both focal adhesion assembly and disassembly (Fig. 3), suggesting that focal adhesion turnover, which requires focal adhesion assembly and disassembly spatially and temporarily, regulates the invasion of cancer cells.

PIPKIγ is a major enzyme that controls polyphosphoinositide metabolism in HCT116 cells. PIPKIγ knockdown results in significant reduction in the level of PI(4,5)P_2_ and decreases PI(3,4,5)P_3_ levels even more substantially (Fig. 8). PI(3,4,5)P_3_ plays an important role in tumorigenesis and cancer metastasis. However, PIPKIγ knockdown does not affect the activation of Akt, a major target of PI(3,4,5)P_3_, in HCT116 cells (data not shown), probably because Akt can be activated by PI(3,4)P_2_, which is not directly affected by PIPKIγ.

Inhibition of PI 3-kinase using LY294002 does not influence focal adhesion formation in HCT116 cells, suggesting that PI(4,5)P_2_ instead of PI(3,4,5)P_3_ is responsible for PIPKIγ-mediated focal adhesion formation. PI(4,5)P_2_ promotes vinculin binding to talin and actin and has been shown to be essential for focal adhesion formation [32]. Our result shows that PIPKIγ regulates vinculin localizing to focal adhesions in HCT116 cells (Fig. 9). Taken together, it is most likely that PIPKIγ regulates focal adhesion formation through PI(4,5)P_2_-mediated vinculin activation.

Cancer invasion is a complicated process, requiring spatial and temporary regulation of cell-matrix adhesions, cell protrusions and matrix-degradation [38]. PI(4,5)P_2_ generated by PIPKIγ regulates cancer invasion through modulating cell adhesion dynamics. Although PI(3,4,5)P_3_ is not essential for focal adhesion formation, it may also play a role in other aspects of cancer invasion, probably through activating Rac [39].

## Materials and Methods

### Reagents

Anti-paxillin antibody (clone 5H11) was from Millipore; Anti-PIPKIγ polyclonal antibody was from Cell Signaling Technology; Anti-tubulin antibody and pLKO1 lentivirus PIPKIγ shRNA (sequence: CCG GGC AGT CCT ACA GGT TCA TCA ACT CGA GTT GAT GAA CCT GTA GGA CTG CTT TTT G) were from Sigma; DyLight 549 conjugated goat anti-mouse IgG (H+L) was from Thermo Scientific; Fibronectin and recombinant HGF were from Akron Biotech; Growth factor reduced Matrigel was from BD Bioscience; The plasmid pEGFP-PIPKIγ was a gift from Dr. Mark Ginsberg (University of California-San Diego); Pfu Ultra was from Agilent Technologies; DNA primers were synthesized by Integrated DNA Technologies.

### Plasmid construction

The plasmid pEGFP-PIPKIγ^W647F^ was generated by pfu Ultra-based PCR using pEGFP-PIPKIγ as template and 5′-GAT GAG AGG AGC TTT GTG TAC TCC CCG CTC-3′/5′-GAG CGG GGA GTA CAC AAA GCT CCT CTC ATC-3′ as primers. pEGFP-PIPKIγ^K188,200R^ and pZZ-PIPKIγ^K188,200R^ were created by PCR using pEGFP-PIPKIγ and pZZ-PIPKIγ [40] as templates, respectively, and sequentially 5′-GAC GAG TTC ATC ATC AGG ACC GTC ATG CAC-3′/5′-GTG CAT GAC GGT CCT GAT GAT GAA CTC GTC-3′ and 5′-GTT CCT GCA GAG GCT GCT CCC TGG CTA C-3′/ 5′-GTA GCC AGG GAG CAG CCT CTG CAG GAA C-3′ as primers. pEGFP-PIPKIγ^D316K^ was constructed by using pEGFP-PIPKIγ as template and 5′-AGT TTC AAG ATC ATG AAG TAC AGC CTG CTG CTG GGC-3′/5′-GCC CAG CAG CAG GCT GTA CTT CAT GAT CTT GAA ACT-3′ as primers. pBabe-EGFP- PIPKIγ and -EGFP-PIPKIγ^K188,200R^ were generated by sequentially digesting pEGFP- PIPKIγ and pEGFP-PIPKIγ^K188,200R^ with Age1, treating with Klenow and digesting with Sal1. The Resulting smaller fragments were subcloned into pBabe-puro vector that had been pre-treated with EcoR1, Klenow and Sal1. pBabe-ZZ-PIPKIγ was made by sequentially digesting pZZ-PIPKIγ with Age1, blunting with Klenow, and digesting with Sal1. The smaller fragments were sub-cloned into pBabe-neo vector that had been treated with BamH1, Klenow, and Sal1. pBabe-ZZ-PIPKIγ^Rescue^ were created by PCR using pBabe-ZZ-PIPKIγ as template and sequentially 5′-GGC ATC ATC GAC ATC CTG CAA TCG TAC AGG TTC -3′/5′-GAA CCT GTA CGA TTG CAG GAT GTC GAT GAT GCC -3′ and 5′- TCG TAC AGG TTG ATA AAG AAA CTG GAG CAC ACC TG-3′/5′- CAG GTG TGC TCC AGT TTC TTT ATC AAC CTG TAC GA-3′ as primers. All plasmids were sequenced by Eurofins MWG Operon (Huntsville, AL).

### Cell culture, transfections and infections

CHO-K1 Chinese hamster ovary cells, HCT116 human colon cancer cells and 293T human embryonic kidney cells were from the American Type Culture Collection and were maintained in DMEM medium (Mediatech, Inc.) containing 10% fetal bovine serum (FBS), penicillin (100 U ml^−1^) and streptomycin (100 µg ml^−1^). CHO-K1 cells were transfected with Safectine RU50 (Syd Labs) according to the manufacturer's protocol. HCT116 cells that stably express EGFP-PIPKIγ WT, or –PIPKIγ^K188, 200R^, were obtained by transfecting the cells with TurboFect (Fermentas) and sorting EGFP-positive cells after G418 selection in the University of Kentucky Flow Cytometry Facility, or by infecting with pBabe retrovirus and selecting with puromycin. HCT116 cells stably expressing shRNA control or PIPKIγ shRNA were obtained by infecting with pLKO1 lentivirus and selected with puromycin.

### Preparation of viruses and cell infection

The 293T cells were transfected with pBabe retrovirus or pLKO1 lentivirus system using Safectine RU50 transfection reagent according to the manufacturer's protocol. The medium of transfectants was collected at 48 and 72 h, filtered through 0.45-µm filter and concentrated by ultracentrifugation. The virus particles were applied to overnight cultures of HCT116 cells for infection. Cells stably expressing recombinant proteins were obtained by growing infected cells in the presence of 1 mg/ml puromycin for 10 days.

### Immunofluorescence staining and TIRF imaging

Cells were plated on glass coverslips that were precoated with 5 µg/ml fibronectin. For immunofluorescence staining, the cells were fixed with 4% paraformaldehyde permeabilized with 0.5% Triton X-100, blocked with 5% BSA in PBS, and then incubated with anti-paxillin mAb. Paxillin was then visualized by incubating with DyLight 549 Conjugated goat Anti-mouse IgG (H+L). Paxillin staining and EGFP fluorescence were viewed by using a Nikon Eclipse Ti TIRF microscope equipped with a 60×, 1.45 NA objective, CoolSNAP HQ2 CCD camera (Roper Scientific). Images were acquired and analyzed by using NIS-Elements (Nikon). To quantify the number and area of focal adhesions, paxillin immunofluorescence images were thresholded to include only focal adhesions and the number and area were calculated by using the software.

### Time-lapse live fluorescence imaging

CHO-K1 cells that stably express EGFP-PIPKIγ WT, or -PIPKIγ^K188, 200R^ were transfected with mDsRed-paxillin. At 24 h post-transfection, the cells were trypsinized and plated on MatTek dishes (with a glass coverslip at the bottom) that had been precoated with fibronectin (5 µg ml^−1^). The cells were cultured for 3 hours and TIRF images were taken using the Nikon Eclipse Ti TIRF microscope stated earlier and the temperature and humidity were maintained by using a INU-TIZ-F1 microscope incubation system (Tokai Hit). Images were recorded at 1-min intervals for a 120 min period. Focal adhesion assembly and disassembly rate constants were analyzed as described previously [22].

### PIPKIγ activity assays

pZZ-PIPKIγ and pZZ-PIPKIγ^K188,200R^ were transfected into CHO-K1 cells. At 24 h post-transfection, the cells were harvested in a lysis buffer (50 mM Tris-HCl, pH8.1, 140 mM NaCl, 50 mM NaF, 1% Triton X-100, 10 mM 2-mercaptoethanol, 0.5 mM AEBSF,10 µg/ml aprotinin, 10 µg/ml leupeptin, 5 µg/ml E-64, 5 µg/ml pepstatin, 5 µg/ml bebstatin,). Cell lysates were cleared by centrifugation and pZZ-PIPKIγ and pZZ-PIPKIγ^K188,200R^ in supernatants were immuno-precipitated using IgG-Agarose beads. The beads were washed three times with lysis buffer and washed once with a kinase buffer (50 mM Tris-HCl, pH7.5, 5 mM MgCl2, 25 mM KCl, 0.5 mM EGTA, and 0.5 mM ATP). The beads were incubated with 100 µL of the kinase buffer containing 100 µM PI(4)P for 30 min at 37°C. PI(4,5)P_2_ formed in these assays was extracted using modified Bligh-Dyer extraction [41]. Phosphoinositides were quantitated using a Shimadzu UFLC coupled with an ABI 4000-Qtrap hybrid linear ion trap triple quadrupole mass spectrometer in multiple reaction monitoring (MRM) mode. 17:0–20:4 PI(4)P, 17:0–20:4 PI(4,5)P_2_ and 17:0–20:4 PI(3,4,5)P_3_ (Avanti Polar Lipids) were used as internal standards. PIP, PIP2 and PIP3s were analyzed on an XTerra C8, 3.5 u, 3.0×100 mm column. Detailed experimental procedures are described in Materials S1.

### Quantitation of Polyphosphoinositides in cells

Polyphosphoinositides were extracted using a modified Bligh-Dyer extraction [41] and were derivatized using trimethylsilyl diazomethane as described [42]. Polyphosphoinositides were measured as their TMS-diazomethane derivatives using a Shimadzu UFLC equipped with a Vydac 214MS C4, 5 µ, 4.6×250 mm column, coupled with an ABI 4000-Qtrap hybrid linear ion trap triple quadrupole mass spectrometer in multiple reaction monitoring (MRM) mode. 17:0–20:4 PI(4)P, 17:0–20:4 PI(4,5)P_2_ and 17:0–20:4 PI(3,4,5)P_3_ were used as internal standards. Detailed experimental procedures are described in Materials S1.

### Centrifugation assays

HCT116 cells growing on 60-mm dishes were incubated in 2 ml of 2 µg/ml calcein-AM in opti-MEM for 20 min at 37°C. The Cells were then trypsinized, washed and re-suspended in normal growth media containing 10%FBS. The cell suspensions (100 µl, 40,000 cells/well) were added to a 96-well plate coated with different concentrations of fibronectin, centrifuged at 180g in a Beckman Coulter Allegra X-15R centrifuge (SX4750A rotor) and allowed to attach for 1h in 37°C CO2 incubator. Each well was then carefully aspirated to remove floating cells and refilled with fresh PBS-dextrose. An initial fluorescence (F_0_) was read to determine the density of cells before detachment using a Promega Glomax multi+ Detection system (490 nm excitation, 510–570 nm emission). The lid was then removed, and the plate was covered with sealing tape and centrifuged upside down at 150 g for 5 min to detach the cells. The wells were carefully aspirated and refilled with fresh PBS-dextrose. The fluorescence after centrifuging (F_a_) was read to determine the density of cells that remain attached.

### Invasion Assays

One hundred microliters of Matrigel (1:30 dilution in serum-free DMEM medium) was added to each Transwell polycarbonate filter (6 mm diameter, 8 µm pore size, Costar) and incubated with the filters at 37°C for 4 h. HCT116 cells were trypsinized and washed 3 times with DMEM containing 1% FBS. The cells were resuspended in DMEM containing 1% FBS at a density of 1×10^6^ cells/ml. The cell suspensions (100 µl) were seeded into the upper chambers, and 600 µl of DMEM medium containing 1% FBS and 5 µg/ml Fibronectin with or without 50 ng/ml HGF were added to the lower chambers. The cells were allowed to invade for 36 h in a CO2 incubator. The invaded cells were fixed for 15 min with 3.7% formaldehyde and stained using 0.1% crystal violet in 10% ethanol for 30 min. The number of invaded cells per field was counted under a light microscope at ×200.

## Supporting Information

Figure S1 **PIPKIγ^D316K^, a kinase-dead mutant, was unable to promote focal adhesion formation in CHO-K1 cells.** (A) TIRF images of CHO-K1 cells expressing PIPKIγ or PIPKIγ^D316K^. Cells were transiently transfected with pEGFP-PIPKIγ WT or –PIPKIγ^D316K^, and then stained for paxillin. Scale bar, 20 µm. (B) PIPKIγ^D316K^ was deficient in promoting an increase in focal adhesion number (n = 10, error bar = mean ± s.e.m; P<0.005). (C) Area distribution of focal adhesions in cells expressing PIPKIγ and PIPKIγ^D316K^.
(TIF)

Figure S2 **Neither expression of PIPKIγ^K188,200R^ nor depletion of PIPKIγ impaired the migration of HCT116 cells.** (A) Expression of PIPKIγ^K188,200R^ slightly enhanced the migration of HCT116 cells. The cells that stably express EGFP-PIPKIγ or –PIPKIγ^K188,200R^ were plated on 35 mm MatTek glass bottom dishes coated with 5 µg/ml fibronectin, and grown to 90% confluency. The medium was then changed to DMEM containing 1%FBS and 10 ng/ml HGF for 6 h. A wound was made on the confluent monolayer, and time-lapse cell migration was recorded using a Nikon Biostation IMQ. The pictures were extracted from time-lapse movies. (B) Quantification of the migration speed of HCT116 cells that stably express PIPKIγ or PIPKIγ^K188,200R^ using NIS-Elements AR 3.2. (n = 4, *P<0.05). (C) Depletion of PIPKIγ by using shRNA had no significant effect on the migration of HCT116 cells. The migration of cells stably expressing shRNA control or PIPKIγ shRNA were examined in the absence and presence of HGF (50 ng/ml) by Transwell migration assays. In brief, transwell polycarbonate filters (6 mm diameter, 8 µm pore size, Costar) were coated with fibronectin (5 µg/ml) over night. HCT116 cells were trypsinized and washed 3 times with DMEM containing 1% FBS. The cells were resuspended in DMEM containing 1% FBS at a density of 1×10^6^ cells/ml. The cell suspensions (100 µl) were seeded into the upper chambers, and 600 µl of DMEM medium containing 1% FBS and 5 µg/ml Fibronectin with or without 50 ng/ml HGF were added to the lower chambers. The cells were allowed to migrate for 24 h in a CO2 incubator. The migrated cells were fixed for 15 min with 3.7% formaldehyde and stained using 0.1% crystal violet in 10% ethanol for 30 min. The number of migrated cells per membrane was counted under a light microscope at ×200. (D) Quantification of the migration of HCT116 cells that stably express shRNA control or PIPKIγ shRNA. n = 3, error bar = mean ± s.e.m, P>0.05.
(TIF)

Materials S1 **Quantitation of phosphoinositides by HPLC ESI tandem Mass Spectrometry.**
(DOCX)
