# Supplementary figures and images for: PIPKIγ Regulates Focal Adhesion Dynamics and Colon Cancer Cell Invasion
Source: PLoS One. 2011 Sep 12;6(9):e24775. doi: 10.1371/journal.pone.0024775 (PMC3171478; doi:10.1371/journal.pone.0024775)

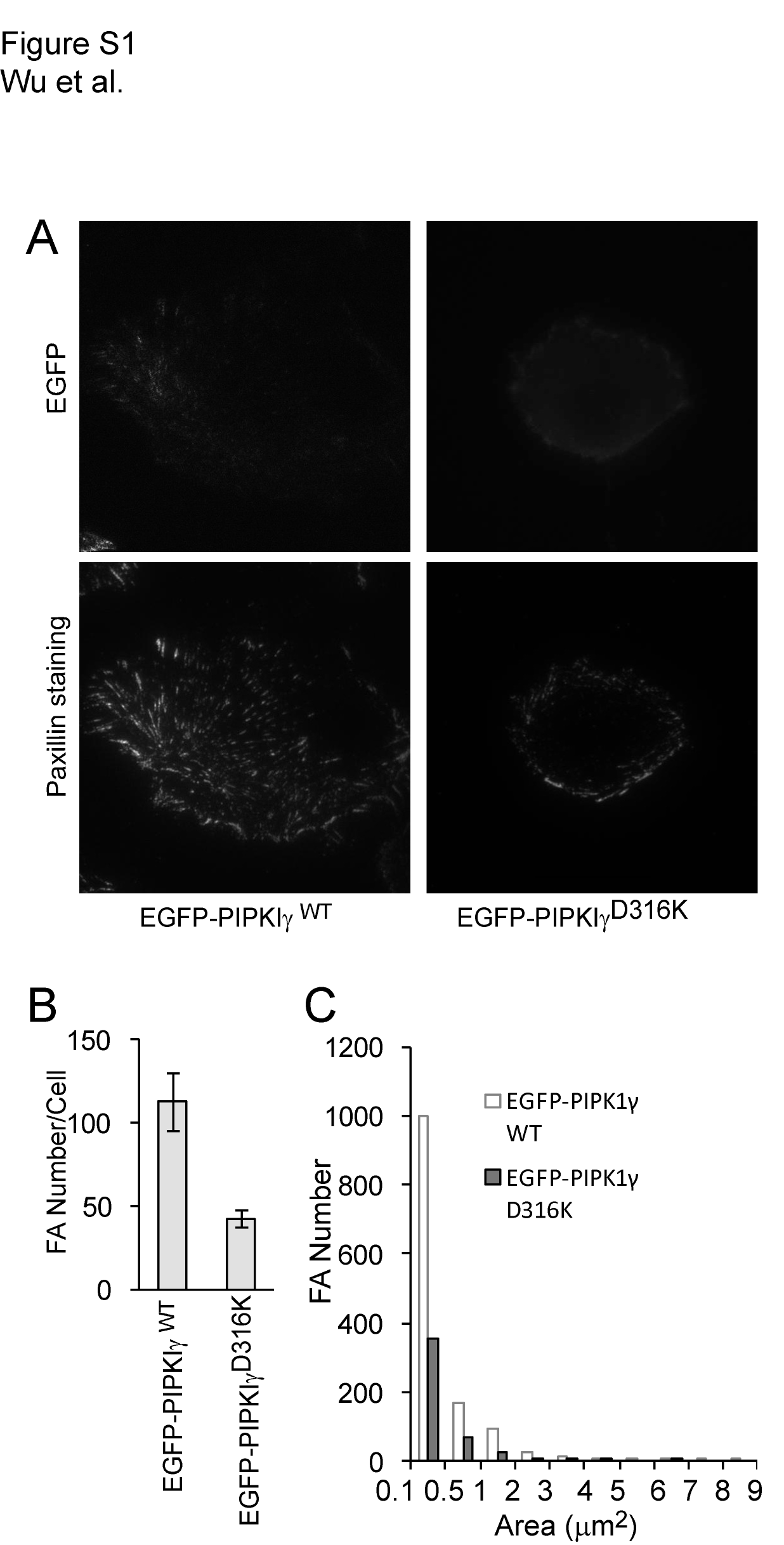

Supplement: Figure S1 — PIPKIγD316K, a kinase-dead mutant, was unable to promote focal adhesion formation in CHO-K1 cells. (A) TIRF images of CHO-K1 cells expressing PIPKIγ or PIPKIγD316K. Cells were transiently transfected with pEGFP-PIPKIγ WT or –PIPKIγD316K, and then stained for paxillin. Scale bar, 20 µm. (B) PIPKIγD316K was deficient in promoting an increase in focal adhesion number (n = 10, error bar = mean ± s.e.m; P<0.005). (C) Area distribution of focal adhesions in cells expressing PIPKIγ and PIPKIγD316K. (TIF) [file pone.0024775.s001.tif]

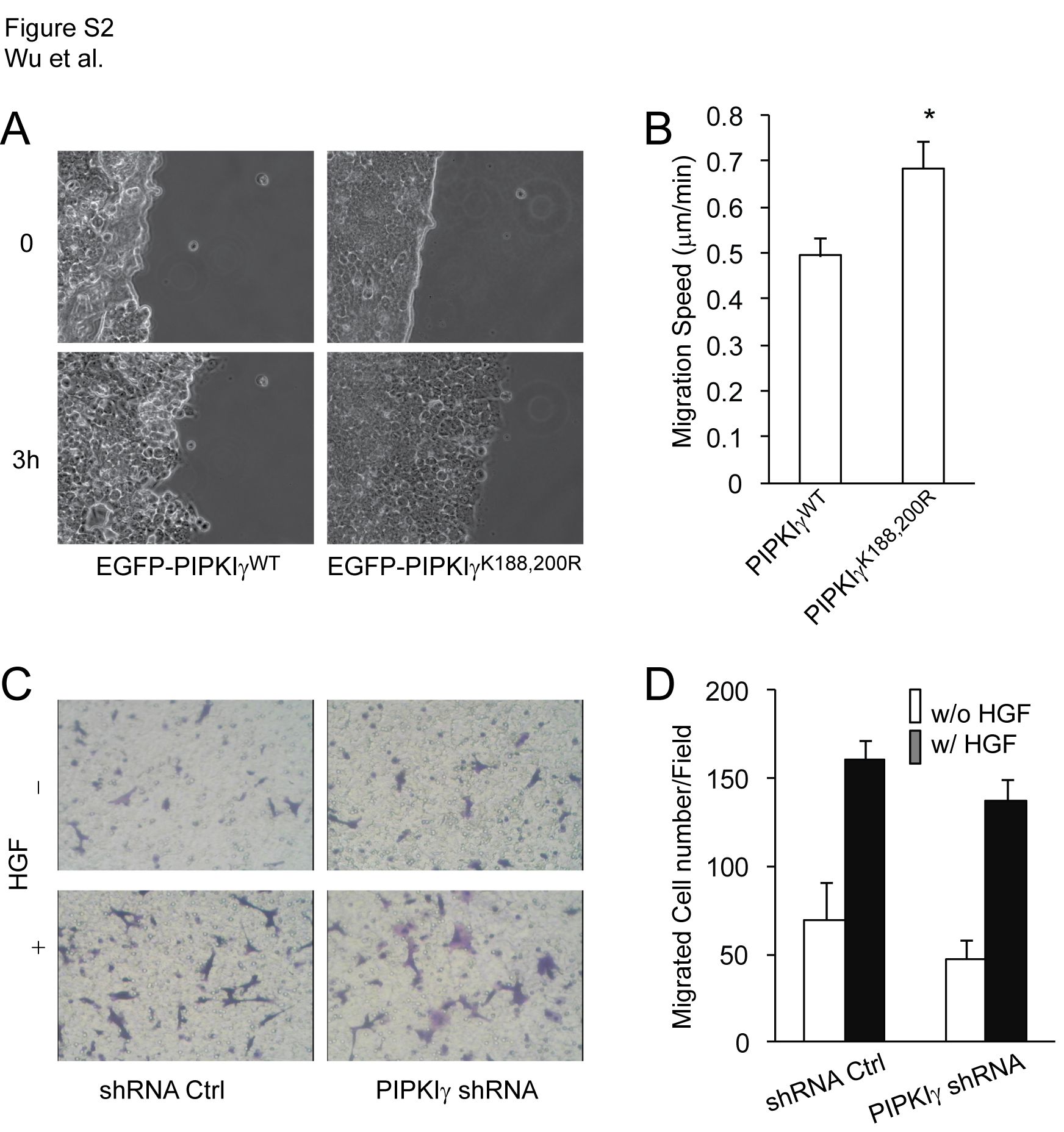

Supplement: Figure S2 — Neither expression of PIPKIγK188,200R nor depletion of PIPKIγ impaired the migration of HCT116 cells. (A) Expression of PIPKIγK188,200R slightly enhanced the migration of HCT116 cells. The cells that stably express EGFP-PIPKIγ or –PIPKIγK188,200R were plated on 35 mm MatTek glass bottom dishes coated with 5 µg/ml fibronectin, and grown to 90% confluency. The medium was then changed to DMEM containing 1%FBS and 10 ng/ml HGF for 6 h. A wound was made on the confluent monolayer, and time-lapse cell migration was recorded using a Nikon Biostation IMQ. The pictures were extracted from time-lapse movies. (B) Quantification of the migration speed of HCT116 cells that stably express PIPKIγ or PIPKIγK188,200R using NIS-Elements AR 3.2. (n = 4, *P<0.05). (C) Depletion of PIPKIγ by using shRNA had no significant effect on the migration of HCT116 cells. The migration of cells stably expressing shRNA control or PIPKIγ shRNA were examined in the absence and presence of HGF (50 ng/ml) by Transwell migration assays. In brief, transwell polycarbonate filters (6 mm diameter, 8 µm pore size, Costar) were coated with fibronectin (5 µg/ml) over night. HCT116 cells were trypsinized and washed 3 times with DMEM containing 1% FBS. The cells were resuspended in DMEM containing 1% FBS at a density of 1×10^6^ cells/ml. The cell suspensions (100 µl) were seeded into the upper chambers, and 600 µl of DMEM medium containing 1% FBS and 5 µg/ml Fibronectin with or without 50 ng/ml HGF were added to the lower chambers. The cells were allowed to migrate for 24 h in a CO2 incubator. The migrated cells were fixed for 15 min with 3.7% formaldehyde and stained using 0.1% crystal violet in 10% ethanol for 30 min. The number of migrated cells per membrane was counted under a light microscope at ×200. (D) Quantification of the migration of HCT116 cells that stably express shRNA control or PIPKIγ shRNA. n = 3, error bar = mean ± s.e.m, P>0.05. (TIF) [file pone.0024775.s002.tif]
