# Supplementary material for: PIPKIγ Regulates Focal Adhesion Dynamics and Colon Cancer Cell Invasion
Source: PLoS One. 2011 Sep 12;6(9):e24775. doi: 10.1371/journal.pone.0024775 (PMC3171478; doi:10.1371/journal.pone.0024775)
Supplement: Materials S1 — Quantitation of phosphoinositides by HPLC ESI tandem Mass Spectrometry. (DOCX) [file pone.0024775.s003.docx]

**Supporting information** (PONE-D-11-13195R1)

**Fig. S1 PIPKIγ^D316K^, a kinase-dead mutant, was unable to promote focal adhesion formation in CHO-K1 cells.** (A) TIRF images of CHO-K1 cells expressing PIPKIγ or PIPKIγ^D316K^. Cells were transiently transfected with pEGFP-PIPKIγ WT or –PIPKIγ^D316K^, and then stained for paxillin. Scale bar, 20 μm. (B) PIPKIγ^D316K^ was deficient in promoting an increase in focal adhesion number (n=10, error bar=mean ± s.e.m; P <0.005). (C) Area distribution of focal adhesions in cells expressing PIPKIγ and PIPKIγ^D316K^.

**Fig. S2 Neither expression of PIPKIγ^K188,200R^ nor depletion of PIPKIγ impaired the migration of HCT116 cells.** (A) Expression of PIPKIγ^K188,200R^ slightly enhanced the migration of HCT116 cells. The cells that stably express EGFP-PIPKIγ or –PIPKIγ^K188,200R^ were plated on 35mm MatTek glass bottom dishes coated with 5 µg/ml fibronectin, and grown to 90% confluency. The medium was then changed to DMEM containing 1%FBS and 10ng/ml HGF for 6 h. A wound was made on the confluent monolayer, and time-lapse cell migration was recorded using a Nikon Biostation IMQ. The pictures were extracted from time-lapse movies. (B) Quantification of the migration speed of HCT116 cells that stably express PIPKIγ or PIPKIγ^K188,200R^ using NIS-Elements AR 3.2. (n=4, *P<0.05). (C) Depletion of PIPKIγ by using shRNA had no significant effect on the migration of HCT116 cells. The migration of cells stably expressing shRNA control or PIPKIγ shRNA were examined in the absence and presence of HGF (50 ng/ml) by Transwell migration assays. In brief, transwell polycarbonate filters (6 mm diameter, 8 µm pore size, Costar) were coated with fibronectin (5 μg/ml) over night. HCT116 cells were trypsinized and washed 3 times with DMEM containing 1% FBS. The cells were resuspended in DMEM containing 1% FBS at a density of 1×10^6^ cells/ml. The cell suspensions (100 µl) were seeded into the upper chambers, and 600 µl of DMEM medium containing 1% FBS and 5 μg/ml Fibronectin with or without 50 ng/ml HGF were added to the lower chambers. The cells were allowed to migrate for 24 h in a CO2 incubator. The migrated cells were fixed for 15 min with 3.7% formaldehyde and stained using 0.1% crystal violet in 10% ethanol for 30 min. The number of migrated cells per membrane was counted under a light microscope at ×200. (D) Quantification of the migration of HCT116 cells that stably express shRNA control or PIPKIγ shRNA. n=3, error bar=mean ± s.e.m, P>0.05.

**Materials S1 Quantitation of phosphoinositides by HPLC ESI tandem Mass Spectrometry**

For *in vitro* measurements of PIPKIγ activity (Fig 2A), phosphoinositides were extracted from the assay mixture using modified Bligh-Dyer extraction[41]. Phosphoinositides were quantitated using a Shimadzu UFLC coupled with an ABI 4000-Qtrap hybrid linear ion trap triple quadrupole mass spectrometer in multiple reaction monitoring (MRM) mode. 17:0-20:4 PI(4)P, 17:0-20:4 PI(4,5)P_2_ and 17:0-20:4 PI(3,4,5)P_3_ (Avanti Polar Lipids) were used as internal standards. PIP, PIP2 and PIP3s were analyzed on an XTerra C8, 3.5 μ, 3.0 X 100 mm column. The mobile phase consisted of 50:50 methanol: water with 0.13% of 70% TEA (in methanol) as solvent A and isopropanol with 0.13% 70% TEA (in methanol) as solvent B. The flow rate was 0.5 mL/min with 0% solvent B for 2 min, then gradually increased to 90% B over the next 7 min and maintained at 90% B for the last 1 min. Column was equilibrated to initial conditions for 3 min. Column temperature was maintained at 30 °C. The sample injection volume was 10 μL. The mass spectrometer was operated in positive electrospray ionization mode with optimal ion source settings determined by synthetic standards of 17:0-20:4 PI(4)P, 17:0-20:4 PI(4,5)P_2_ and 17:0-20:4 PI(3,4,5)P_3_ with a declustering potential of 161 V, entrance potential of 10 V, collision energy of 27 V, collision cell exit potential of 22 V, curtain gas of 20 psi, ion spray voltage of 5500 V, ion source gas1/gas2 of 40 psi and temperature of 550 °C. Precursor ion masses employed for selected reaction monitoring corresponded to the protonated mass of the indicated phosphoinositide molecular species with a product ion of m/z 401 used for quantitation of PIP_2_s and m/z 321 for quantitation of various PIPs.

For quantitation of phosphoinositides in lipid extracts from cultured cells (Fig 8) we used methods adapted from those reported by Clark et al 2011. Lipids were extracted from monolayer cultures of cells using modified Bligh-Dyer extraction and were derivatized directly in the lower phase of these extractions by reaction with trimethylsilyl diazomethane. Samples were reconstituted with 4:1 methanol: water prior to analysis. Polyphosphoinositides were measured as their methylated derivatives using a Shimadzu UFLC coupled with an ABI 4000-Qtrap hybrid linear ion trap triple quadrupole mass spectrometer in multiple reaction monitoring (MRM) mode. 17:0-20:4 PI(4)P, 17:0-20:4 PI(4,5)P_2_ and 17:0-20:4 PI(3,4,5)P_3_ were used as internal standards to monitor recovery throughout the process. PIP, PIP_2_ and PIP_3_s were analyzed on a Vydac 214MS C4, 5 μ, 4.6 X 250 mm column. The mobile phase consisted of 0.1 % TFA in water as solvent A and 0.1 % TFA in acetonitrile as solvent B. The flow rate was 0.5 mL/min with 75% solvent B for 5 min, then gradually increased to 100% B over the next 5 min, maintained at 100% B for 10 min, brought back to initial 75% B over next 5 min and maintained at 75% B for last 5 min. Column temperature was maintained at 30 °C. The sample injection volume was 10 uL. The mass spectrometer was operated in the positive electrospray ionization mode with optimal ion source settings determined by TMS-diazomethane derivatized synthetic standards of 17:0-20:4 PI(4)P, 17:0-20:4 PI(4,5)P_2_ and 17:0-20:4 PI(3,4,5)P_3_ with a declustering potential of 161 V, entrance potential of 10 V, collision energy of 27 V, collision cell exit potential of 22 V, curtain gas of 20 psi, ion spray voltage of 5500 V, ion source gas1/gas2 of 40 psi and temperature of 550 °C. MRM transitions monitored for 18:0-20:4 PIP_3_, 18:0-20:4 PIP2, 18:0-20:4 PIP were 1225.57/627.6, 1117.22/627.6 and 1009.57/627.6 respectively where Q1 mass represents the protonated methylated PIP mass and the Q3 mass corresponds to the diacylglycerol fragment of the parent ion. MRM ion transitions for 17:0-20:4 PI(4)P, 17:0-20:4 PI(4,5)P_2_ and 17:0-20:4 PI(3,4,5)P_3_ were 996.671/614.2, 1104.635/614.5 and 1212.623/614.5 respectively. Other species measured were as follows; 18:0-20:3 PIP_3_:1227.59/629.6;18:0-20:3 PIP_2_:1119.59/629.6; 18:0-18:2 PIP_3_: 1201.57/603.53;18:0-18:2 PIP_2_:1093.58/603.53;18:0-18:1 PIP_3_:1203.59/605.6;18:0-18:1 PIP_2_:1095.59/605.6; 18:0-20:2: PIP_3_1229.6/631.56;18:0-20:2 PIP_2_:1121.6/631.56;18:0-18:2; PIP:877.57/603.53;18:0-18:1PIP:879.59/605.6;18:0-20:2 PIP:905.6/631.56; 18:0-20:3 PIP:1011.59/629.6. Note that these methods do not provide information about the enantiomeric configuration of the phosphoinositide headgroup and cannot therefore distinguish between positional isomers of the different phosphoinositides. PIP, PIP_2_ and PIP_3_s were analyzed on an XTerra C8, 3.5 μ, 3.0 X 100 mm column. The mobile phase consisted of 50:50 methanol: water with 0.13% of 70% TEA (in methanol) as solvent A and isopropanol with 0.13% 70% TEA (in methanol) as solvent B. The flow rate was 0.5 mL/min with 0% solvent B for 2 min, then gradually increased to 90% B over the next 7 min and maintained at 90% B for the last 1 min. Column was equilibrated to initial conditions for 3 min. Column temperature was maintained at 30 °C. The sample injection volume was 10 uL. The mass spectrometer was operated in the positive electrospray ionization mode with optimal ion source settings determined by synthetic standards of 17:0-20:4 PI(4)P, 17:0-20:4 PI(4,5)P_2_ and 17:0-20:4 PI(3,4,5)P_3_ with a declustering potential of 161 V, entrance potential of 10 V, collision energy of 27 V, collision cell exit potential of 22 V, curtain gas of 20 psi, ion spray voltage of 5500 V, ion source gas1/gas2 of 40 psi and temperature of 550 °C. Precursor mass measured in an MRM ion transition corresponded to the protonated mass while 401 was a common product ion for measuring various PIP_2_s and 321 for various PIPs.
